# A Novel CNN Model for Classification of Chinese Historical Calligraphy Styles in Regular Script Font

**DOI:** 10.3390/s24010197

**Published:** 2023-12-29

**Authors:** Qing Huang, Michael Li, Dan Agustin, Lily Li, Meena Jha

**Affiliations:** 1 School of Education and the Arts, Central Queensland University, Rockhampton, QLD 4701, Australia; 2 School of Engineering and Technology, Central Queensland University, Rockhampton, QLD 4701, Australia; m.li@cqu.edu.au (M.L.); l.li@cqu.edu.au (L.L.); m.jha@cqu.edu.au (M.J.); 3 Centre of Railway Engineering, School of Engineering and Technology, Central Queensland University, Rockhampton, QLD 4701, Australia; d.agustin@cqu.edu.au

**Keywords:** deep learning, convolutional neural network (CNN), Chinese calligraphy, styles classification, handwriting recognition

## Abstract

Chinese calligraphy, revered globally for its therapeutic and mindfulness benefits, encompasses styles such as regular (Kai Shu), running (Xing Shu), official (Li Shu), and cursive (Cao Shu) scripts. Beginners often start with the regular script, advancing to more intricate styles like cursive. Each style, marked by unique historical calligraphy contributions, requires learners to discern distinct nuances. The integration of AI in calligraphy analysis, collection, recognition, and classification is pivotal. This study introduces an innovative convolutional neural network (CNN) architecture, pioneering the application of CNN in the classification of Chinese calligraphy. Focusing on the four principal calligraphy styles from the Tang dynasty (690–907 A.D.), this research spotlights the era when the traditional regular script font (Kai Shu) was refined. A comprehensive dataset of 8282 samples from these calligraphers, representing the zenith of regular style, was compiled for CNN training and testing. The model distinguishes personal styles for classification, showing superior performance over existing networks. Achieving 89.5–96.2% accuracy in calligraphy classification, our approach underscores the significance of CNN in the categorization of both font and artistic styles. This research paves the way for advanced studies in Chinese calligraphy and its cultural implications.

## 1. Introduction

Chinese calligraphy is a revered art form with a rich history dating back thousands of years, and it is considered a valuable cultural heritage of the world. To date, tens of millions of people around the globe collect or practice this art form. Additionally, there are numerous historical calligraphy works that require authentication for commercial purposes in the art collection and auction market, as well as in the field of culture study and research. Moreover, practitioners of calligraphy require evaluation methods to aid in the study and practice of calligraphy styles and aesthetics. Consequently, the automatic recognition of calligraphy styles through image processing techniques holds great significance in art collections, auctions, and academic pursuits.

**Figure 1:**
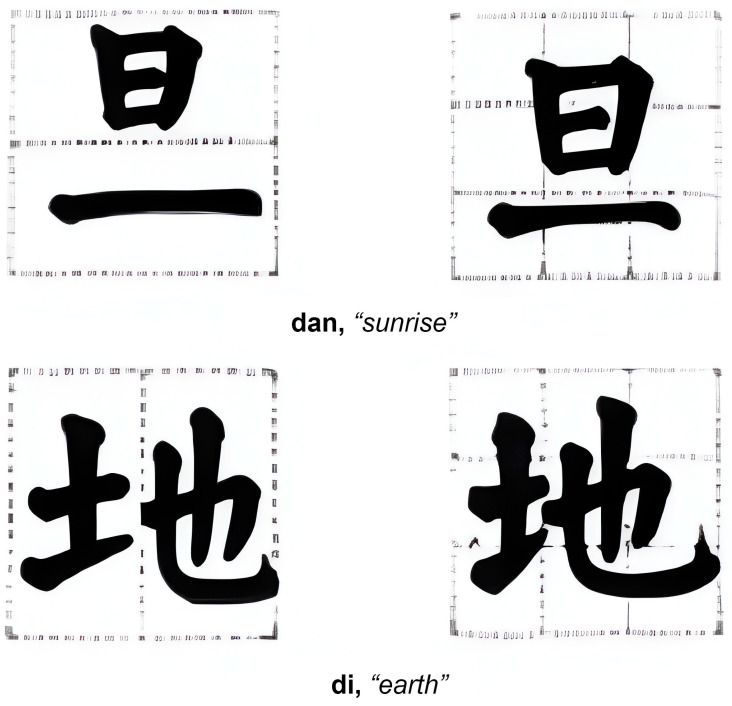
(**Left**): the split down the middle yields a sense of unbalance. (**Right**): the well-developed character measured by the Nine-Palaces grid from Fitzgerald [2].

**Figure 2:**
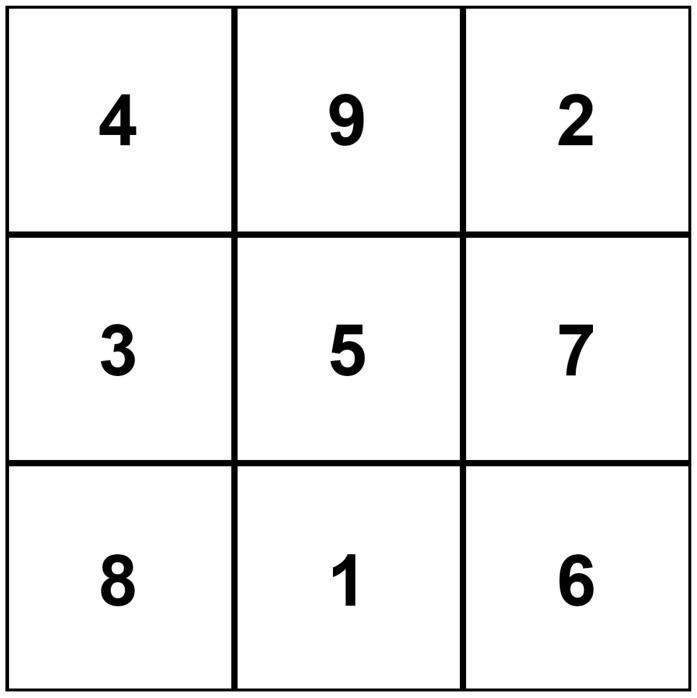
The root structure of the Nine-Palaces grid (Wong [3]).

Our previous research works have inspired us to explore the use of the notion of fractal dimension from modern Chaos theory to measure Chinese artworks [1], providing a quantifiable measurement in explaining Chinese unique aesthetic principles. Our research has revealed that, by tradition, Chinese calligraphy employs a unique ancient way of measurement to maintain visual balance in each Chinese character (Figure 1). This so-called Nine-Palaces grid (Figure 2) is frequently used to evaluate the beauty of calligraphy works in Chinese. The grid system is also utilized in practicing Chinese calligraphy to help practitioners develop their sense of balance and beauty in writing. We contend that such a grid system approach is akin to the 3 × 3 convolution filter, or kernel, employed in convolutional neural networks (CNNs) to detect the underlying traits and features of any visual pattern.

Encouraged by the discovery, we decided to use convolutional neural network (CNN) methods to further analyze Chinese calligraphy works, since classical CNN methods [4] have already carried out a very accurate job in recognizing the human handwriting of digits such as those trained by the MNIST dataset. The recent development of computer imageries, data science, and deep learning shows a surge of interest in image digitizing and character recognition in the field of machine learning and convolutional neural network research and application. Utilizing CNN for the recognition of Chinese handwriting calligraphy characters becomes a novel one. Chinese calligraphy has a rich history of historical works and calligraphers. Numerous scholars and followers have studied, admired, and copied the Chinese calligraphy styles. As we research through all the current literature in the field, many works have already been carried out in applying CNN in Chinese calligraphy studies. However, most of the studies focus on the classification of five basic fonts, namely, seal, clerical, cursive, semi-cursive, and regular (Table 1). These fonts visually look distinctive from each other and were appropriated and developed through various Chinese historical periods. They are rather a broader categorization. In fact, even within each of these font categories, numerous historical calligraphers have their unique personal styles and flavors that differentiate them from each other and reflect their distinctive individual personalities, feelings, spiritual, and intellectual levels.

Our main research question is “does the CNN can recognize unique personal calligraphy styles even from within the same font type such as a standard regular script (kai shu) of Chinese calligraphy?”. To investigate this research question, we collected and developed a dataset of four famous historical calligraphers, spanning the Chinese Tang dynasty (690–907 A.D.) period, who are considered to have appropriated the regular script style within the period [5]. Tang dynasty calligraphy styles share some of the common features of the regular script style, with each having a unique characteristic of its own, being considered as reaching the highest aesthetic level of their calligraphy styles [5], and being set as an exemplar for next generations. It is from these calligraphers of the Chinese Tang dynasty period that the regular script has been established and used for empirical official documents. After developing the dataset, we then trained and developed a calligraphy recognition system based on a convolutional neural network model. The system can recognize and differentiate the four historical personal calligraphy styles with a great accuracy rate. We implemented a calligraphy style dataset to train the network, and then used the trained system to evaluate any given amateur works and test the system’s feasibility. Our experimental results show that the system is reliable in evaluating the personal progress of the learning styles of calligraphy. The major goal of this study was to investigate practical approaches to increase image categorization accuracy while keeping the execution time of the application program under control.

## 2. Related Works

A large amount of research has been carried out in the field of calligraphy recognition and classification. Li [6] proposed a support vector machine-based method in 2013 with a recognition rate of 96% for the official and regular scripts, while Lin [7] presented a location- and hashing-based method in 2014 with a recognition rate of 81%, 90%, 100%, 81%, and 63% for seal, official, regular, running, and cursive scripts, respectively. In 2014, Mao [8] developed a method based on using Gabor filters as a texture discriminator with a recognition rate of 99%, 98%, 100%, 51%, and 71% for seal, official, regular, running, and italic fonts, while Wang et al. [9] carried out a principal component analysis-based method in 2016 with a recognition rate of 99%, 96%, 91%, 73%, and 24% of seal, official, regular, running, and italic fonts. Yan [10] developed a local pixel probability pooling-based method in 2018 with a recognition rate of 92.4% of official, regular, and running fonts. Cui [11] designed a system for recognizing Chinese calligraphy and its font using context images based on a multi-level convolutional neural network. 

Utilizing a CNN for recognizing and classifying calligraphy works, Chen [12] combined the CNN approach with traditional recognition algorithms to identify the calligraphy image and the corresponding Chinese characters. Liu [13] also proposed a CNN approach to identify a specific historical calligrapher in Chinese history for authentication purposes. Zhai [14] used a deep neural network model to extract the inscription from paintings and focused on identifying calligraphy and painting regions. Li [15] used CNN to recognize different traditional Chinese calligraphy styles and achieved a test accuracy of 88.6%. Wen and Sigüenza [16] proposed a CNN-based method for Chinese calligraphy style recognition based on a full-page document. Wang et al. [17] proposed an automatic evaluation approach for Chinese calligraphy based on disk B-spline curve (DBSC) vectorization of characters, iterative closest point (ICP) registration between handwritten and standard character skeletons, and comprehensive shape similarity metrics calculated on both the global character and individual strokes. Gao et al. [18] proposed using deep convolutional neural network features with modified quadratic discriminant function classification for automatic Chinese calligraphic style recognition, showing a significant improvement in performance over global and local features named as scale invariant feature transform (SIFT) descriptors and achieving 99.8% on the standard calligraphic character library (SCL) and 94.2% on the Chinese calligraphy dataset (CCD) with the modified quadratic discriminant function (MQDF). Zou et al. [19] proposed combining the softmax cross-entropy loss with an average variance similarity ranking loss to increase the convolutional neural network accuracy in recognizing handwritten Chinese characters from 93.79% with softmax alone to 95.58%, a 1.79% absolute improvement. Zhang et al. [20] made a significant contribution in their research by developing a CNN model enhanced with a convolutional block attention module (CBAM) and a 5 × 5 convolution kernel, achieving an improved classification of Chinese calligraphy styles with high accuracy. 

Among the studies mentioned, the predominant focus has been on distinguishing the five primary styles of Chinese calligraphy, namely, seal, official, regular, running, and cursive. These research efforts have primarily been aimed at recognizing and classifying these fundamental script styles. Additionally, some studies have explored practical applications, such as the extraction of specific scripts from images, emphasizing the versatility of calligraphy recognition techniques beyond mere classification. 

Our literature review extends to other domains of machine learning that employ advanced CNN models for varied disciplinary objectives. For instance, Liu et al. [21] developed TokenHPE (head pose estimation), a novel transformer-based model for head pose estimation. TokenHPE effectively utilizes orientation tokens to capture complex facial relationships, demonstrating state-of-the-art performance. Liu et al. [22] also developed an innovative approach for head pose estimation using the ARHPE+ model. This model employs a Lorentz distribution learning scheme for a more accurate facial feature extraction in infrared images. Moreover, their team [23] proposed an innovative head pose estimation method using an anisotropic angle distribution learning (AADL)-based convolutional neural network. Their approach leverages covariance pooling layers to capture second-order image features and employs the Kullback–Leibler divergence for robust label prediction. Furthermore, Xu et al. [24] delivered an exhaustive analysis of AI’s role in revolutionizing classical civil engineering across an infrastructure’s full-life cycle, from smart design to disaster response, by combining data-driven machine learning with conventional engineering concepts. The authors in [25] also proposed a nested attribute-based few-shot meta-learning approach that significantly improves structural damage identification, as evidenced by a comprehensive assessment on a dataset featuring 1000 examples across 10 different damage categories, achieving an impressive accuracy of 93.5% and an AUC of 0.96. Their method, which necessitates fewer samples while providing enhanced adaptability and a balanced precision–recall ratio for various damage types, advances our comprehension of CNN applications and offers fresh insights that could inform our methodology.

## 3. A CNN Model for the Classification of Chinese Calligraphy Images

In this section, we provide a concise introduction to the functioning of convolutional neural networks (CNNs) in the context of image analysis, a topic central to our study. The CNN concept is inspired by Hubel and Wiesel’s discovery [26] of the visual perception structure in the visual cortex of animals, in which cells detect light in receptive fields. Neocognition [27], a computational model based on local neuron connectivity and hierarchically organized image transformations, was proposed as a primitive CNN. LeCun et al. established the modern CNN framework in a study carried out in 1998 [4]. Since 2006, research has focused on training challenges, shifting to deeper structures. Notable works include AlexNet [28,29], VGGNet [30], GoogLeNet [31], and ResNet [32], reflecting a trend towards deeper architectures for enhanced performance.

A typical convolutional neural network (CNN) comprises three types of fundamental layers: convolutional layers, pooling layers, and fully connected layers. The convolutional layer plays a pivotal role in feature extraction by utilizing multiple filter kernels to compute diverse feature maps from the input image or the previous layer’s feature map. These filters traverse the input image, engaging in a mathematical operation known as convolution. During this process, each element-wise filter multiplies its values with the corresponding pixels in the input image, and subsequently aggregates the results to yield a singular output. By applying a range of filters to the input image, the convolutional layer can discern various patterns and features. When multiple convolutional layers are employed, the network can progressively learn and represent multi-level abstractions of the original image. Mathematically, the *k*-th feature map at the *l*-th layer can be computed using the following convolutional operations [33]:

(1) $$z_{i,j,k}^{l}=\sum_{\alpha \;=\;0}\sum_{\beta \;=\;0}w_{\alpha ,\beta ,k}^{l}x_{i+\alpha ,j+\beta }^{l}+b_{k}^{l}$$

where *w* and *b* are the weight matrix and bias term at the *l*-th layer, respectively, and *x* is the input image patch (or the previous layer’s feature map) centered at location (*i*,*j*). After the convolutional operations, an activation function is applied to introduce nonlinearity to the CNN to produce an activation map. This can be expressed as follows:

(2) $$a_{i,j,k}^{l}=\varphi (z_{i,j,k}^{l})$$

where *φ* (·) is the activation function. The most common activation function is the rectified linear unit (ReLU) function [34].

As the dimension of the activation maps is typically very large, down sampling is necessary to reduce the size of the activation map. This process is crucial not only for managing the computational load during network training, but also for diminishing the count of training samples associated with the majority class. The mechanism of down sampling is realized through the pooling operation, strategically implemented within the pooling layers of the convolutional neural network (CNN). Mathematically, the pooling operation can be succinctly expressed as follows:

(3) $$y_{i,j,k}^{l}=f_{pool}(a_{m,n,k}^{l})\;\;\;\;\;\;\;\forall (m,n)\in R_{ij}$$

where *y* is the output of the pooling operation associated with the *k*-th feature map, *f_pool_*(·) denotes the pooling function, and *R_ij_* is a local neighboring around the location (*i*,*j*). The popular pooling operations in applications are max pooling or average pooling [33].

After traversing multiple convolutional and pooling layers, the resultant feature map undergoes flattening, serving as input for one or more fully connected layers within the CNN. It is within these fully connected layers that the CNN engages in high-level reasoning. These layers transform the 2D feature map into a one-dimensional feature vector. In the context of a classification task, this derived vector is then passed through a softmax function, assigning it to a specific category.

**Figure 3:**
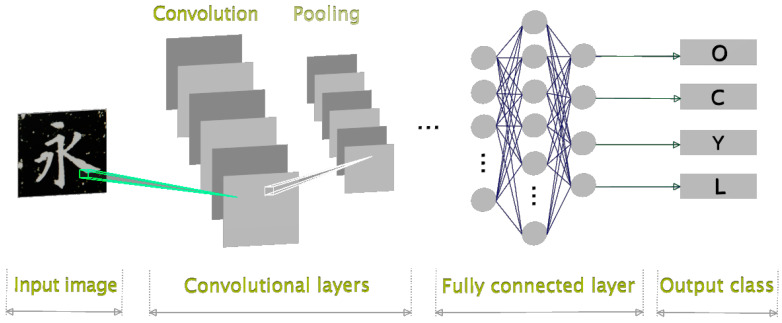
The proposed style-oriented CNN architecture for Chinese calligraphy classification. (The import image example in the diagram is the Chinese character of “永” meaning eternity.)

Figure 3 illustrates the proposed style-oriented CNN architecture designed for Chinese calligraphy classification. In this framework, the input image comprises a Chinese character extracted from a curated collection of stylized fonts. The network’s output corresponds to the categories representing the distinctive styles of calligraphers. The labels O, C, Y, and L denote Ouyang Xun, Chu Cunliang, Yan Zhengqin, and Liu Gongquan, respectively.

To train a CNN network, it is necessary to define an objective function, which is also known as a loss function that evaluates how well the network will perform during training and testing. For the image classification task, the cross-entropy loss is the most commonly used loss function. This function calculates the difference between the predicted class probabilities and the true class labels. Mathematically, the cross-entropy loss function can be expressed as follows [35]:

(4) $$f^{t}\left(W\right)=-\sum_{i=1}^{m}\tilde{y_{i}}\log\left(\mathrm{softmax}\left(w_{i}x\right)\right)\;=-\sum_{i=1}^{m}\tilde{y_{i}}\log\left(y_{i}\right)$$

where *m* represents the total number of classes, *y*_i_ denotes the *i*-th prediction class, *ŷ_i_* is the *i*-th true class of training samples, and *w*_i_ is the weight vector.

A dataset comprising images and labels is required to train the CNN network in order to resolve the image classification problem. The network employs a learning algorithm to adjust its free parameters and to achieve the desired output. During training, the loss function acts as a guide for CNN, helping it learn to make more accurate predictions by minimizing the error between predicted and true outputs. The dataset we used for this specific problem includes images of calligraphy work, similar to handwritten digits or handwritten English characters but featuring Chinese words converted into images, with four distinct categories, namely, Ouyang Xun (O), Chu Suiliang (C), Yan Zhenqing (Y), and Liu Gongquan (L). The Chinese characters vary in complexity based on their texture, shape, and other factors such as stroke angles, amount, thickness, etc. In the next section, we offer a detailed description of the numerical experiments that we have carried out using the CNN model with suitable architecture parameters on our specific dataset.

## 4. Experiments and Analysis

### 4.1. Dataset Construction

Acquiring a large and comprehensive dataset is critical to model the complexity of accurately classifying calligraphy styles. The training accuracy of the model related to epistemic uncertainty can be enhanced using more data. Epistemic uncertainty refers to the uncertainty of the model and is often due to a lack of training data. Unfortunately, in our previous experiments, we were unable to find appropriate data for our research. As a result, we recognized the importance of constructing our own dataset to focus on epistemic uncertainty. We constructed a new dataset named the Chinese calligraphy dataset CQU (CCD-CQU).

The preparation of the dataset was a time-consuming process as we had specific target calligraphers from a particular period: Tan dynasty (690–970 A.D.). The Chinese calligraphy works we used for our study were sourced from a public website that had an encyclopedic collection of scanned and digitized pictures of Chinese calligraphy works from different authors and periods (http://www.yac8.com) (accessed on 15 August 2021). The Chinese calligraphy works were identified according to the targeted author works and were sorted chronologically.

For Ou Yangxun (557–641 A.D.), we focused on his famous inscription work “Jiu Cheng Gong Li Quan Ming “ stele (《九成宫醴泉铭》), which is considered one of the finest representatives of his calligraphy art. It was originally an essay documenting the trip of emperor Tang Taizong (599–649 A.D.). His calligraphy style is often regarded as strict, neat, and well-organized, making it a popular choice for calligraphy teachers to assign to their students to copy from as their first example.

For Chu Suiliang (596–658 A.D.), we collected works not only from famous inscription stele sources such as “Yan Ta Sheng Jiao Xu” (《雁塔圣教序》), “Meng Fa Shi Bei Ming” (《孟法师碑铭》), and “Qian Zi Wen Bei” (《千字文碑》), but also some rare collections of his actual writings on paper, such as “Ying Fu Jing” (《阴符经》). These works spanned his lifetime and are representative of his calligraphy style.

For Yan Zhenqing (709-785 A.D.), we selected the main body of characters from his famous inscription stele “Duo Bao Ta Bei” (《多宝塔碑》) and included his alleged actual writings on paper, such as “Zhu Shan Lian Ju” (《竹山连句》). Yan has over 138 legacy works, and “Duo Bao Ta Bei” was written when he was at the peak of his career and writing style.

For Liu Gongquan (778–865 A.D.), we chose the main body of calligraphy characters from the famous “Mysterious Tower Stele” (《玄秘塔碑》), which is considered the masterpiece of his calligraphy art and often the first choice for followers to copy and practice the regular script style, as well.

**Figure 4:**
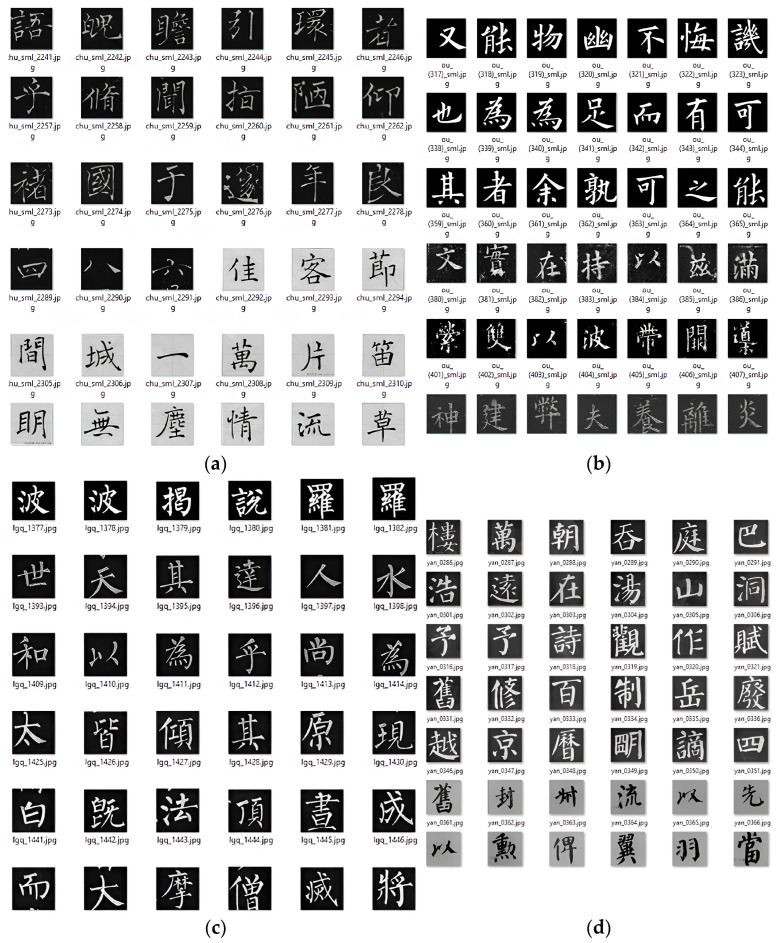
(**a**) Chu Suiliang calligraphy examples. (**b**) Ou Yangxun calligraphy examples. (**c**) Liu Gongquan calligraphy examples. (**d**) Yan Zhengqing calligraphy examples.

We also included miscellaneous examples from other copybooks that extracted individual characters from various historical documents or copied from inscriptions for followers to study. We collected over 2000 images of the calligraphy characters for each author. We processed them in Photoshop by cropping, resizing, and reducing them into 64 × 64 pixels in dimension and in black and white images (Figure 4a–d) so that it would be faster for CNN training and testing. There are characters which are repeated and have differences in value, contrast, variations, and noises in the background. The assumption we worked on was that these nuances were not significant for the learning algorithm and probably would be even more beneficially challenging for deep learning.

### 4.2. Numerical Experiments, Results, and Discussion

**Figure 5:**
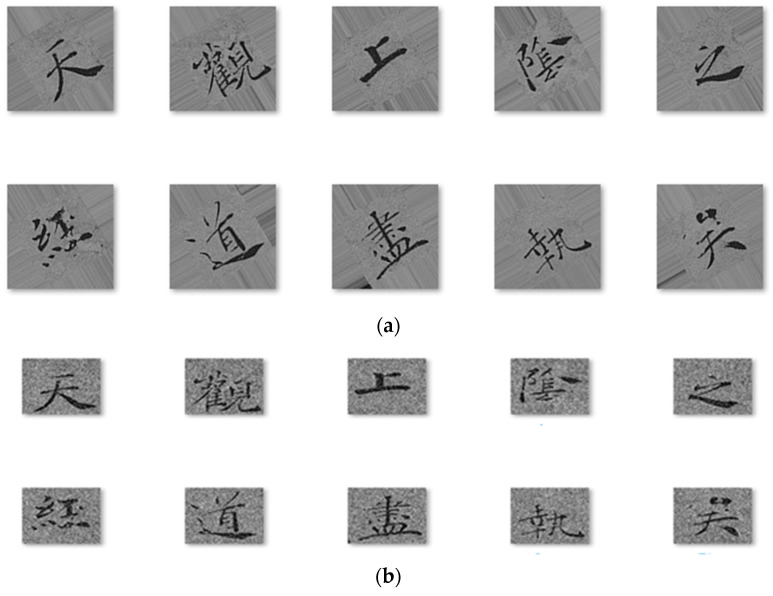
(**a**) Rotated image data samples. (**b**) Image data samples with the addition of random background noise.

To improve the performance of the neural network model, it is a common practice to train the model with more data so that uncertainty can be captured. Data augmentation has been typically used to obtain additional training samples by applying transformations such as flipping, cropping, rotating, scaling, and elastic deformations to the dataset samples. Therefore, we have added an equal amount of image data through data augmentation procedures. These procedures involve generating 50% of augmented images through rotations with angles ranging from 10 to 180 degrees, with uniform distribution intervals of 10 degrees. The remaining 50% of image data were generated by adding a random background. Figure 5a,b illustrate some samples of augmented data images. Overall, there were 16,564 images processed in the experiment.

The behavior of CNNs is highly complex. In the studied classification problem using the CNN method, the dataset is the base where the built network requires learning. On the other hand, the performance accuracy of a CNN model for a specific learning task is significantly influenced by its architecture and algorithmic parameters [36]. To ensure the best possible accuracy, we carefully studied and tuned the hyperparameters of our CNN model, resulting in the design of five different architectures for our application. The key parameters of our architecture are summarized in Table 2, whereas Table 3 (1)–(5) outline the detailed configuration manners for filters of varying sizes, which are referred to as configuration types 1 to 5 in the following discussions.

As a CNN model comprises iterative blocks of convolutional layers and pooling layers, the combination of convolution and pooling operations can vary significantly. Each specific combination of convolution and pooling operations can be treated as a configuration of a distinct architecture for the network in the numerical experiments. For instance, AlexNet [29], a well-known CNN model, employs a 11 × 11 convolution filter with a stride of 4 as the initial layer, followed by a 5 × 5 filter, and subsequently uses a 3 × 3 filter for all other convolutional layers. The VGG model [30], on the other hand, employs two continuous convolutional layers followed by a pooling layer, repeated twice, and then follows with three continuous layers plus a pooling layer, with each convolution operation using a 3 × 3 filter.

In our design, configuration types 1, 2, and 4 use convolution filter sizes of 3 × 3, 5 × 5, and 7 × 7, respectively. Configuration type 3 uses a 5 × 5 filter size for the first convolution, followed by all others using a 3 × 3 filter size. Configuration type 5 follows a VGG-like style with a total of seven convolutional layers. Each configuration starts with a certain number of filters (K), with the number of filters doubling at the subsequent convolution layer. The five configurations have a total number of 11, 9, 11, 9, and 15 layers, respectively. In addition to the architecture parameters, we have included the algorithm-related parameters in Table 4.

In this study, the main objective was to explore feasible ways to maximize image classification accuracy, while ensuring that the application program’s running time remains manageable. Given a set of fixed algorithm-related parameters, the performance accuracy of a CNN model can be defined approximately as a function of the architecture parameters (*F*, *K*, *C*, *N*) as follows:

(5) a=f(F,K,C,N)

where *F* is the filter size, *K* is the number of filters, *C* represents the configuration manner, and *N* represents the number of neurons at the fully connected layers (which may comprise two or more components).

**Figure 6:**
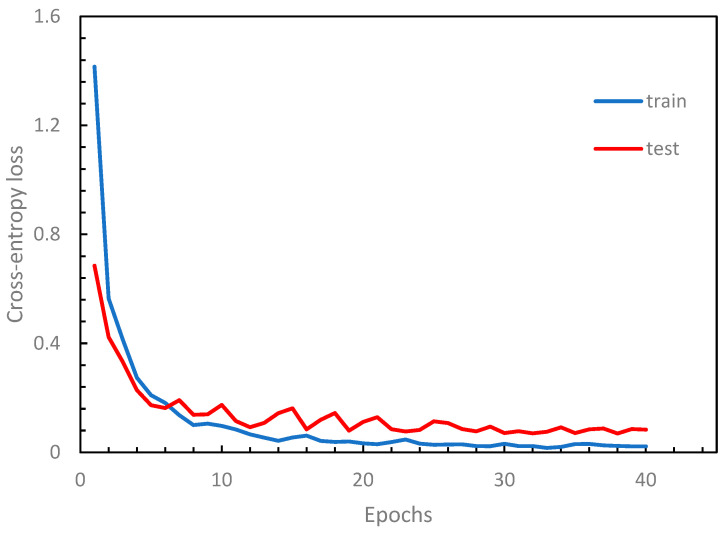
Cross-entropy loss curves.

**Figure 7:**
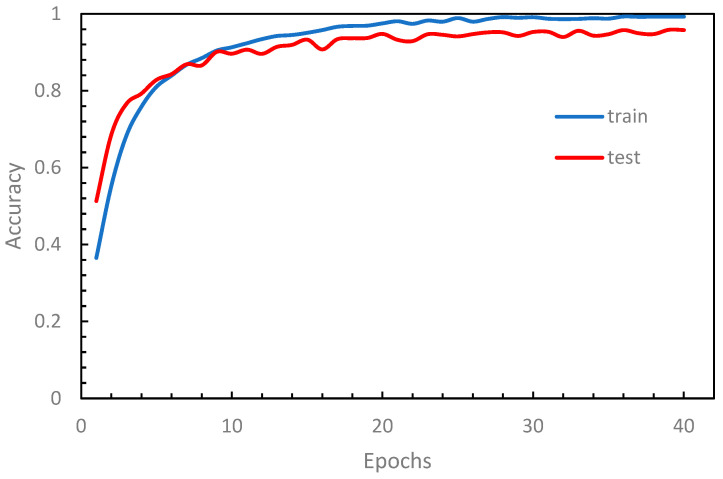
Accuracy curves.

We implemented a Python application program using the TensorFlow library [37] based on the design described above. To evaluate the performance of our model, we conducted numerical experiments on our dataset, which was divided into 80% for training and 20% for testing. The results of a typical training example are shown in Figure 6, where the loss function tends to stabilize after 25 epochs, indicating a convergence in the training process. Figure 7 illustrates the performance curves against the epoch, where both the training and testing accuracy curves approach stability after 25 epochs. The running time for each modification on an ordinary PC with a single GPU ranges between approximately 12 min and 45 min, depending on the configuration type and the number of filters (K). The VGG-like architecture takes a longer running time due to its deeper structure.

**Figure 8:**
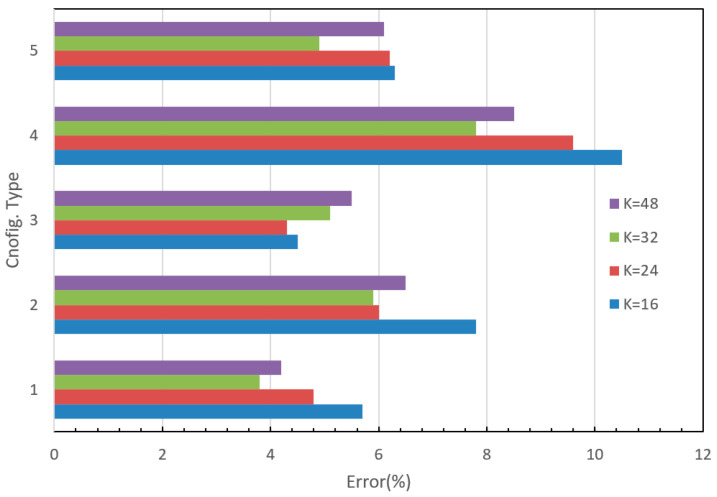
Performance errors for various configurations.

**Figure 9:**
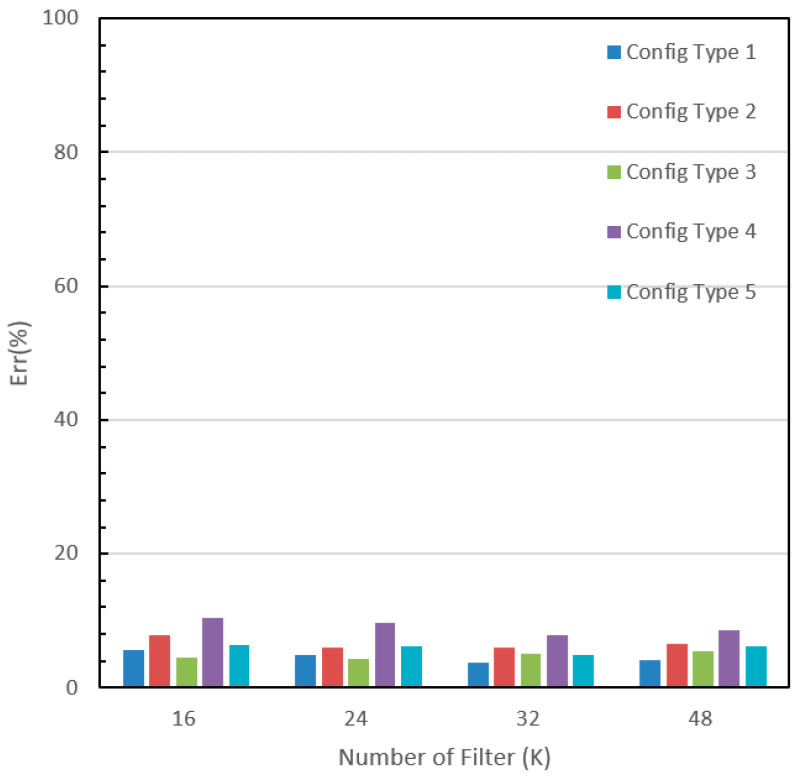
Performance errors vs. the number of filter (K).

The recorded accuracies for each modification are presented in Table 5. The accuracy on the training dataset varied from 93.7% to 99.5%, which indicates that the designed networks are well trained. On the other hand, the accuracies on the testing dataset ranged between 89.5% and 96.2%. Figure 8 depicts the test error graph for different configuration types and starting numbers of filters (K). Configuration type 1 with K = 32 yielded the lowest error rate of 3.8%, which is more in line with human recognition abilities for this type of image. The graph reveals that for configuration type 1 which has a small filter size (3 × 3), the test error decreases as the number of filters increases, but it reaches a saturation point at K = 32. Configuration types 1 and 3 have lower error rates, while configuration types 2 and 4 have relatively high error values. For a small-size image input, a smaller filter size achieves better accuracy, while a larger filter size may result in some information loss in the extracted feature maps. Furthermore, configuration type 5 is a VGG-like style configuration with a deeper structure, but it did not produce an impressive accuracy as expected. This could be attributed to the fact that the additional convolutional operations may not necessarily extract more meaningful features for this particular image dataset. Moreover, as illustrated in Figure 9, the optimal number of filters for most configurations should be 24 or 32. The experimental results have clearly demonstrated that the built network with the carefully tuned architecture parameters can correctly recognize the different font styles, i.e., categories in our dataset. The outstanding performance accuracy is attributed to the correct extraction of font style features. A Chinese character typically comprises eight basic components such as dots, horizontals, verticals, hooks, lifts (or raises), curves, throws, and presses, each of which has a dynamic corresponding form known as sideways, tighten, bow or strive, leap, whip, sweep, peck, and hack. Different calligraphers apply these basic writing forms in unique ways, including variations in motion direction, graphic manner, stroke method, and the force applied to create their works. For example, Ou preferred using sharp and bold strokes, whereas Chu often intentionally handled the strokes, lines, and dots with turning points to reflect an aesthetic and abstract value that transcends the characters’ physical appearance. These special writing manners result in individual font styles and their features in the image are reflected in texture, shape, angle, and pattern. These features are effectively extracted by convolutional operations using various filters in a series of feature maps, which leads to an accurate classification. Increasing the size of the training dataset in future work is likely to further improve prediction accuracy.

Table 6 presents the selected test accuracies of individual classes for configuration types 1 and 2 with K = 32. The results indicate that the different category characters exhibit varying performance accuracy, with class O having the lowest accuracy. This could be attributed to the imbalanced sample data, as the O class has the fewest number of images. Imbalanced training data can lead to several problems that affect classification accuracy [38,39]. If one class has significantly fewer samples than the others, the network may exhibit biased behaviors towards that class and may classify even the least number of sample images as belonging to that corresponding class. The underlying cause is that CNN attempts to minimize overall errors, which may cause it to focus more on the dominant classes and less on the minority classes. Consequently, for the fewer samples in the minority classes, the network may not have sufficient information to learn the features belonging to that class, thus reducing the classification accuracy.

It is noteworthy to point out that the individual Chinese characters within this dataset exhibit a slight degree of blurring. From the perspective of image analysis, this blurring can be regarded as a form of inherent noise, with a potential impact on classification accuracy that may not be substantial. In our ongoing research, we intend to delve deeper into the effects of blurred images on our specific research problem. To accomplish this, we plan to conduct additional experiments that build upon and extend the current study, further refining our understanding of this phenomenon.

**Figure 10:**
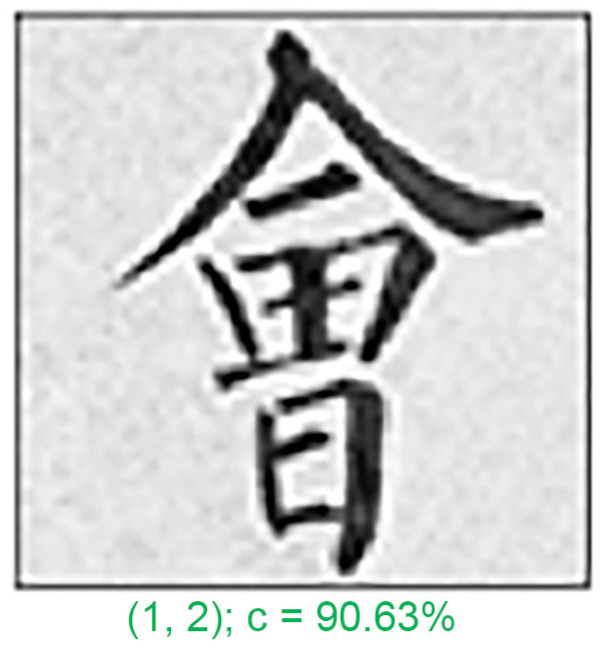
Misclassified example 1 (The Chinese character for “meeting”).

**Figure 11:**
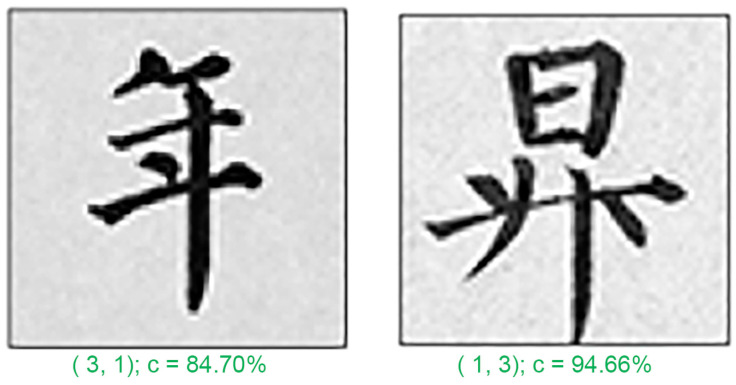
Misclassified example 2. (The Chinese character for “year” and “rise”.)

**Figure 12:**
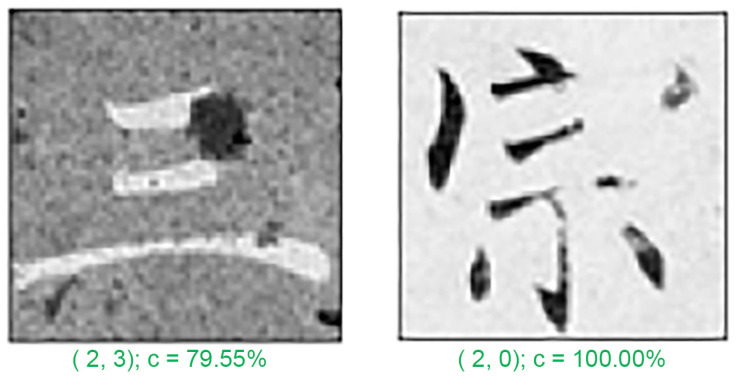
Misclassified example 3. (The Chinese character for “three” and “sect”).

In any classification problem, misclassification is a common issue. In this study, we analyzed a few typical misclassification examples. Figure 10, Figure 11 and Figure 12 depict the five test patterns that our CNN misclassified. Below each image, the correct answer (left) and the network prediction (right) are displayed. The errors were mostly caused by the following reasons:

Firstly, the issue of symmetry can make it difficult for calligraphers to write characters in a distinct order of strokes. For instance, the character “會”, which means meeting (Figure 10), is symmetrical and can easily be written similarly in terms of strokes and order.

Secondly, the stroke width is another factor that can contribute to the difficulty of character recognition. In the examples provided (Figure 11), calligraphers 1 and 3 have stroke widths that are similar in thickness. Additionally, calligraphers 0 and 2 are thinner and elegant, while calligraphers 1 and 3 are thicker and bolder. This similarity in stroke width can confuse the CNN when trying to differentiate between certain characters.

Lastly, the simplicity of some characters, such as character “三”, meaning number three, (Figure 12 right) which only has 2–3 strokes, can make them challenging to differentiate. Additionally, big stains or noise signals in the examples (Figure 12 left) can further hinder the accuracy of CNN detection, resulting in the aforementioned problems.

Overall, these examples provide valuable insights into the challenges faced by CNNs in character recognition and highlight the need for continued improvement in this field.

## 5. Conclusions and Future Works

This study elucidates the utilization of a novel convolutional neural network (CNN) architecture to distinguish among the four primary styles of historical calligraphers from the Tang dynasty (690–907 A.D.). The proposed model exhibits superior performance, achieving accuracy rates between 92% and 98% in Chinese calligraphy classification. This achievement is attributed to the optimal architecture parameters identified during our research. Furthermore, the training dataset curated for this purpose has proven effective and accessible, with potential for expansion through the inclusion of additional examples.

Key contributions of this research include:

- Development of a comprehensive image dataset for Chinese calligraphy (regular script) classification, comprising over 8000 images, serving as a valuable resource for future scholarly endeavors.
- Pioneering the application of CNN in the classification of personal styles in Chinese calligraphy.
- Achieving elevated performance metrics with the CNN model, as evidenced by test accuracy rates ranging from 89.5% to 96.2%.
- Effective fine tuning of both architectural and algorithmic parameters within the classical CNN framework, resulting in the identification of optimal parameters associated with outstanding performance accuracy.
- Preliminary investigation into the challenge of an imbalanced distribution within the training data, laying the groundwork for addressing this issue in subsequent studies.

Enhancements such as data augmentation techniques, including image shifting, rotation, and the introduction of random background noise, have the potential to further refine accuracy. Future experiments will leverage multiple high-performance GPUs to evaluate training durations, currently ranging from 10 to 40 min. A proficiently trained network can swiftly predict classifications for new image data, presenting commercial opportunities such as the development of personal trainer applications for the study, evaluation, and improvement in Chinese calligraphy.

Prospective research avenues might explore the application of this methodology to various script styles and artists’ personal styles or the creation of new datasets for these purposes. Given the complexity of the cursive style, which is often challenging for untrained observers, a CNN-based tool could provide significant support to scholars and practitioners in this artistic domain.

## Figures and Tables

**Table 1:** The Chinese phrase: “Renowned is a mountain not for its height but for the immortal who lives in it” in five basic Chinese calligraphy styles.

*Seal (zhuan):*	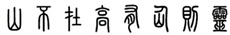
*Clerical (li):*	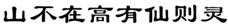
*Cursive (cao):*	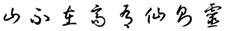
*Semi-cursive (xing):*	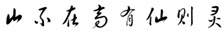
*Regular (kai):*	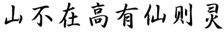

**Table 2:** Critical architecture parameters of our CNN design.

Parameters	Values
Input image size	64 × 64 × 1
Filter size (F × F)	3 × 3, 5 × 5, 7 × 7
Number of filters (K)	16, 24, 32, 48
Pooling size (Max Pooling)	2 × 2 stride = 2
Configuration type (C_n_)	C_1_, C_2_, C_3_, C_4_, C_5_
Neuron numbers of FC Layer (N)	512, 256, 128

**Table 3:** CNN configuration types (1)–(5).

(1) Filter size 3 × 3 (K = 32)			
**Block**	**Layer**	**Layer Type**	**Description** **(Feature Map Size)**
Block 1	L1	Conv + ReLU	32@62 × 62
	L2	Max Pooling	32@31 × 31
Block 2	L3	Conv + ReLU	64@29 × 29
	L4	Max Pooling	64@14 × 14
Block 3	L5	Conv + ReLU	128@12 × 12
	L6	Max Pooling	128@6 × 6
Block 4	L7	Conv + ReLU	256@4 × 4
	L8	Max Pooling	256@2 × 2
Block 5 (FC layers)	L9	FC1	512 neurons
	L10	FC2	256 neurons
	L11	FC3 (softmax)	4 neurons
(2) Filter size 5 × 5 (K = 32)			
**Block**	**Layer**	**Layer Type**	**Description** **(Feature Map Size)**
Block 1	L1	Conv + ReLU	32@60 × 60
	L2	Max Pooling	32@30 × 30
Block 2	L3	Conv + ReLU	64@26 × 26
	L4	Max Pooling	64@13 × 13
Block 3	L5	Conv + ReLU	128@9 × 9
	L6	Max Pooling	128@4 × 4
Block 4 (FC layers)	L7	FC1	512 neurons
	L8	FC2	256 neurons
	L9	FC3 (softmax)	4 neurons
(3) Filter sizes, 5 × 5, 3 × 3 (K = 32)			
**Block**	**Layer**	**Layer Type**	**Description** **(Feature Map Size)**
Block 1	L1	Conv + ReLU	32@60 × 60
	L2	Max Pooling	32@30 × 30
Block 2	L3	Conv + ReLU	64@28 × 28
	L4	Max Pooling	64@14 × 14
Block 3	L5	Conv + ReLU	128@12 × 12
	L6	Max Pooling	128@6 × 6
Block 4	L7	Conv + ReLU	256@4 × 4
	L8	Max Pooling	256@2 × 2
Block 5 (FC layers)	L9	FC1	512 neurons
	L10	FC2	256 neurons
	L11	FC3 (softmax)	4 neurons
(4) Filter size 7 × 7 (K = 32)			
**Block**	**Layer**	**Layer Type**	**Description** **(Feature Map Size)**
Block 1	L1	Conv + ReLU	32@58 × 58
	L2	Max Pooling	32@29 × 29
Block 2	L3	Conv + ReLU	64@23 × 23
	L4	Max Pooling	64@11 × 11
Block 3	L5	Conv + ReLU	128@5 × 5
	L6	Max Pooling	128@2 × 2
Block 5 (FC layers)	L7	FC1	512 neurons
	L8	FC2	256 neurons
	L9	FC3 (softmax)	4 neurons
(5) VGG-like style (filter size 3 × 3, K = 32)			
**Block**	**Layer**	**Layer Type**	**Description** **(Feature Map Size)**
Block 1	L1	Conv + ReLU	32@62 × 62
	L2	Conv + ReLU	32@60 × 60
	L3	Max Pooling	32@30 × 30
Block 2	L4	Conv + ReLU	64@28 × 28
	L5	Conv + ReLU	64@26 × 26
	L6	Conv + ReLU	64@24 × 24
	L7	Max Pooling	64@12 × 12
Block 3	L8	Conv + ReLU	128@10 × 10
	L9	Conv + ReLU	128@8 × 8
	L10	Conv + ReLU	128@6 × 6
	L11	Max Pooling	128@3 × 3
Block 4	L12	Conv + ReLU	256@1 × 1
Block 5 (FC layers)	L13	FC1	256 neurons
	L14	FC2	128 neurons
	L15	FC3 (softmax)	4 neurons

**Table 4:** Algorithm-related parameters.

Optimizer	Adam
Learning rate	1.0 × 10^−4^
Batch size	32, 64
Dropout rate	0.25, 0.5

**Table 5:** The accuracy obtained on our dataset with different architecture parameters.

Architecture		Accuracy	
K	Configuration	Training	Testing
16	C_1_	98.6%	94.3%
	C_2_	96.3%	92.2%
	C_3_	98.7%	95.5%
	C_4_	93.7%	89.5%
	C_5_	96.6%	93.7%
24	C_1_	99.2%	95.2%
	C_2_	98.9%	94.0%
	C_3_	99.0%	95.7%
	C_4_	95.7%	90.4%
	C_5_	98.1%	93.8%
32	C_1_	99.5%	96.2%
	C_2_	98.2%	94.1%
	C_3_	98.9%	94.9%
	C_4_	95.2%	92.2%
	C_5_	98.7%	95.1%
48	C_1_	99.1%	95.8%
	C_2_	97.7%	93.5%
	C_3_	98.3%	94.5%
	C_4_	96.8%	91.5%
	C_5_	98.2%	93.9%

**Table 6:** Individual class accuracy for configuration types 1 and 2 and K = 32.

Class	Type 1 (C_1_)	Type 2 (C_2_)
O	93.8%	92.1%
C	98.3%	96.2%
L	97.0%	95.2%
Y	95.7%	93.0%

## Data Availability

The datasets supporting the conclusions of this study are available upon request from the principal author (q.huang@cqu.edu.au). The authors commit to providing access to the data and materials promptly to researchers with a qualified purpose in accordance with the ethical approval vetting the collection of the data.
